# Prenatal and Childhood Traffic-Related Pollution Exposure and Childhood Cognition in the Project Viva Cohort (Massachusetts, USA)

**DOI:** 10.1289/ehp.1408803

**Published:** 2015-04-03

**Authors:** Maria H. Harris, Diane R. Gold, Sheryl L. Rifas-Shiman, Steven J. Melly, Antonella Zanobetti, Brent A. Coull, Joel D. Schwartz, Alexandros Gryparis, Itai Kloog, Petros Koutrakis, David C. Bellinger, Roberta F. White, Sharon K. Sagiv, Emily Oken

**Affiliations:** 1 Department of Environmental Health, Boston University School of Public Health, Boston, Massachusetts, USA; 2 Channing Laboratory, Brigham and Women’s Hospital, Boston, Massachusetts, USA; 3 Department of Environmental Health, Harvard T.H. Chan School of Public Health, Boston, Massachusetts, USA; 4 Department of Population Medicine, Harvard Medical School and Harvard Pilgrim Health Care Institute, Boston, Massachusetts, USA; 5 Department of Biostatistics, Harvard T.H. Chan School of Public Health, Boston, Massachusetts, USA; 6 Department of Hygiene, Epidemiology, and Medical Statistics, University of Athens Medical School, Athens, Greece; 7 The Department of Geography and Environmental Development, Ben-Gurion University of the Negev, Beer Sheva, Israel; 8 Department of Neurology, Boston Children’s Hospital, Harvard Medical School, Boston, Massachusetts, USA; 9 Center for Environmental Research and Children’s Health, School of Public Health, University of California, Berkeley, Berkeley, California, USA

## Abstract

**Background:**

Influences of prenatal and early-life exposures to air pollution on cognition are not well understood.

**Objectives:**

We examined associations of gestational and childhood exposure to traffic-related pollution with childhood cognition.

**Methods:**

We studied 1,109 mother–child pairs in Project Viva, a prospective birth cohort study in eastern Massachusetts (USA). In mid-childhood (mean age, 8.0 years), we measured verbal and nonverbal intelligence, visual motor abilities, and visual memory. For periods in late pregnancy and childhood, we estimated spatially and temporally resolved black carbon (BC) and fine particulate matter (PM_2.5_) exposures, residential proximity to major roadways, and near-residence traffic density. We used linear regression models to examine associations of exposures with cognitive assessment scores, adjusted for potential confounders.

**Results:**

Compared with children living ≥ 200 m from a major roadway at birth, those living < 50 m away had lower nonverbal IQ [–7.5 points; 95% confidence interval (CI): –13.1, –1.9], and somewhat lower verbal IQ (–3.8 points; 95% CI: –8.2, 0.6) and visual motor abilities (–5.3 points; 95% CI: –11.0, 0.4). Cross-sectional associations of major roadway proximity and cognition at mid-childhood were weaker. Prenatal and childhood exposure to traffic density and PM_2.5_ did not appear to be associated with poorer cognitive performance. Third-trimester and childhood BC exposures were associated with lower verbal IQ in minimally adjusted models; but after adjustment for socioeconomic covariates, associations were attenuated or reversed.

**Conclusions:**

Residential proximity to major roadways during gestation and early life may affect cognitive development. Influences of pollutants and socioeconomic conditions on cognition may be difficult to disentangle.

**Citation:**

Harris MH, Gold DR, Rifas-Shiman SL, Melly SJ, Zanobetti A, Coull BA, Schwartz JD, Gryparis A, Kloog I, Koutrakis P, Bellinger DC, White RF, Sagiv SK, Oken E. 2015. Prenatal and childhood traffic-related pollution exposure and childhood cognition in the Project Viva cohort (Massachusetts, USA). Environ Health Perspect 123:1072–1078; http://dx.doi.org/10.1289/ehp.1408803

## Introduction

Evidence supporting the hypothesis that outdoor air pollution affects the brain was recently reviewed at a National Institutes of Health workshop (Block et al. 2012). Participating scientists concluded that more studies were needed to define critical time windows for pollution effects on cognition, assess factors related to vulnerability to outdoor air pollution (e.g., age at exposure, stress, and socioeconomic status), and define the relation of particle pollution composition to its toxicity.

In a study of urban Boston children 8–11 years of age, childhood exposure to black carbon (BC), a marker of traffic pollution, was associated with lower verbal IQ, nonverbal IQ, and visual memory abilities (Suglia et al. 2008). Neither that study nor recent cross-sectional reports of associations between ambient air pollution exposure and reduced cognitive performance (Calderón-Garcidueñas et al. 2011; Freire et al. 2010; Wang et al. 2009) examined pollution effects across different time windows of development. Exposures in late pregnancy to airborne polycyclic aromatic hydrocarbons (PAHs) predicted lower cognitive development at 3 years of age and lower IQ at 5 years in New York City (Perera et al. 2006, 2009) and poorer nonverbal reasoning at 5 years in Krakow, Poland (Edwards et al. 2010). In a recent meta-analysis of European birth cohorts, prenatal exposure to ambient air pollution was associated with lower psychomotor development in children 1–6 years of age, but not with cognitive development (Guxens et al. 2014). Postnatal exposures were not measured in these studies.

We examined associations of child cognitive outcomes with exposure to traffic-related air pollutants in late pregnancy and childhood. Exposure was assessed in several ways: residential proximity to major roadways, near-residence traffic density, and BC and fine particulate matter (aerodynamic diameter ≤ 2.5 μm; PM_2.5_) estimated at the residence level. Because of the known vulnerability of the prenatal period to neurodevelopmental toxicants (Bellinger 2013), we hypothesized that pregnancy exposure would be more influential than childhood exposure.

## Methods

*Study population*. We studied participants in Project Viva, a longitudinal cohort of 2,128 mother–child pairs enrolled during 1999–2002 at mothers’ initial prenatal visits (median, 9.9 weeks of gestation) at eight locations of Atrius Harvard Vanguard Medical Associates, a multi-specialty group practice in urban and suburban eastern Massachusetts. Study procedures for this cohort have been described previously (Oken et al. 2015). Briefly, we administered health and developmental assessments to mothers and children at in-person visits and through mailed annual questionnaires. A total of 1,110 children completed at least one cognitive assessment at a visit in mid-childhood (mean age, 8.0 years; 80% 7 or 8 years at assessment). After excluding participants lacking exposure information, final sample sizes ranged from 960 to 1,104 (depending on analysis), with 1,109 participants included in at least one analysis. The Institutional Review Board of Harvard Pilgrim Health Care approved the study. All mothers provided written informed consent, and children provided verbal assent at the mid-childhood visit.

*Major roadway proximity and near-residence traffic density*. Mothers reported their residential addresses at study visits and on annual questionnaires. We estimated residential proximity to nearest major roadway and near-residence traffic density at birth and date of the mid-childhood cognitive assessment. Using ArcGIS 10.1 with Street MapTM North America (ESRI, Redlands, CA), aerial photographs, and internet resources, we geocoded locations of each reported residential address and calculated distance to the nearest major roadway (U.S. Census class A1 or A2). To assess exposure patterns over pregnancy and childhood, we also calculated major roadway proximity at study enrollment (median, 9.9 weeks gestation) and in early childhood (median, 3.3 years).

Near-residence traffic density was estimated as the length of all roads (kilometers) within 100 m of the residence multiplied by traffic counts on those roads (vehicles/day) [as in Zeka et al. (2008)], using traffic count data published by the Massachusetts Department of Transportation through the Office of Geographic Information (MassGIS 2014). We used 2002 traffic data in estimates for birth addresses, and 2009 traffic data in estimates for mid-childhood addresses.

*Air pollutant exposure assessment*. We estimated residence-specific BC and PM_2.5_ exposures using validated spatiotemporal land use regression models. We previously reported methodologies for these models (Gryparis et al. 2007; Kloog et al. 2012; Zanobetti et al. 2014). Briefly, the BC model was based on daily BC measurements from a central monitoring site on the roof the Harvard Countway Library of Medicine and 148 permanent and temporary BC monitors operating in the region between January 1999 and August 2011. Other inputs included area land use, traffic density, and meteorological data (Gryparis et al. 2007; Zanobetti et al. 2014).

The PM_2.5_ model incorporated satellite aerosol optical depth measurements at the 10 × 10 km grid scale for years 2000–2010 from the Moderate Resolution Imaging Spectroradiometer aboard the Earth Observing System satellites. Additional inputs to the PM_2.5_ model included daily PM_2.5_ concentration measurements from U.S. Environmental Protection Agency and Interagency Monitoring of Protected Visual Environments networks, along with data on area and point sources of PM_2.5_, land use, locations of major roads, and meteorology (Kloog et al. 2012).

Using geocoded residential addresses, we estimated mean BC and PM_2.5_ exposures for the third trimester of pregnancy (188th day after last menstrual period to birth), the year before each child’s cognitive assessment, and the first 6 years of life (birth to 6 years). We assigned exposures only if participants resided in areas where model predictions were available (eastern Massachusetts for the BC model, New England for the PM_2.5_ model) for ≥ 90% of days in an exposure period.

*Cognitive outcomes*. Trained Project Viva staff administered assessments of cognitive development at in-person visits when children were 6.6–10.9 (mean, 8.0) years. We assessed verbal and nonverbal intelligence using the Kaufman Brief Intelligence Test (KBIT-2) (Kaufman and Kaufman 2004), visual motor performance with the Visual-Motor subtest of the Wide Range Assessment of Visual Motor Abilities (WRAVMA) (Adams and Sheslow 1995), and visual memory (design memory and picture memory) with the Visual Memory Index of the Wide Range Assessment of Memory and Learning (WRAML2) (Sheslow and Adams 2003). Assessments were double-scored using published scoring guidelines and supplementary guidelines developed by a pediatric neuropsychologist to ensure consistency among scorers (Project Viva 2002). Staff administering and scoring assessments had no knowledge of participants’ traffic exposure status. Scaled scores were standardized to mean (± SD) of 100 ± 15 for KBIT-2 and WRAVMA, and 10 ± 3 for WRAML2 design memory and picture memory subscores, based on published reference data (Adams and Sheslow 1995; Kaufman and Kaufman 2004; Sheslow and Adams 2003). Neurobehavioral functional domains measured by each of the cognitive assessments are outlined in the Supplemental Material, Table S1.

*Covariates*. Covariate data were collected from study questionnaires, interviews, and children’s medical records. We calculated median annual household income for census tract of residence at the date of cognitive assessment using data from the 2000 United States Census (U.S. Census Bureau 2000). We assessed support for cognitive development in the child’s home using the Home Observation for Measurement of the Environment–Short Form (HOME-SF). HOME-SF scores range from 0 to 22; higher scores represent better support (Frankenburg and Coons 1986). We assessed mothers’ IQ with the KBIT-2. A subset of mothers (*n* = 571) provided blood samples in mid-pregnancy, which were analyzed for lead (Perkins et al. 2014). We accessed clinical blood lead levels in early childhood from medical records for 419 child participants.

*Statistical analyses*. We assessed correlations among exposures, outcomes, and covariates with Spearman correlation coefficients and assessed collinearity among model covariates by calculating variance inflation factors. We ran separate multivariate linear regression models to estimate exposure–outcome associations of each cognitive outcome (verbal IQ, nonverbal IQ, visual motor, design memory, and picture memory) with each exposure type (major roadway proximity, traffic density, BC, and PM_2.5_) for each exposure window (birth and date of testing for traffic measures, third trimester of pregnancy, birth–6 years, and year before testing for BC and PM_2.5_). We analyzed major roadway proximity in three categories using cut points drawn to reflect the exponential-type pattern of spatial decay observed for some components of traffic-related air pollution: < 50 m, 50 to < 200 m, and ≥ 200 m (Hart et al. 2009; Karner et al. 2010; Zhu et al. 2002). We natural log (ln)–transformed traffic density and modeled it as a continuous exposure; BC and PM_2.5_ were also treated as continuous exposures. We scaled effect estimates by the interquartile range (IQR) of ln(traffic density), BC, or PM_2.5_ for the relevant period. We checked our assumption of linearity of the continuous exposure–outcome relationships by fitting generalized additive models with penalized spline smooth terms for continuous exposures, adjusted for all relevant covariates (see below) and visually assessing plotted splines.

We ran initial models minimally adjusted for child sex and age at cognitive testing. Primary models were adjusted for a range of covariates selected as potential confounders based on prior evidence, according to directed acyclic graph theory (Hernán et al. 2002). Covariates for primary models included characteristics of the child [sex, age at cognitive testing, breastfeeding duration (months up to 12), blood lead in early childhood (micrograms per deciliter)], mother [IQ, parity (0, 1, ≥ 2), age at enrollment (< 25, 25–34, ≥ 35 years), marital/cohabitation status (yes/no), education (≥ college graduate/< college graduate), race/ethnicity (black, white, Hispanic, Asian, other), smoking status (never, former, smoked during pregnancy), exposure to secondhand smoke during pregnancy (< 1 hr /≥ 1 hr/week), blood lead in pregnancy (micrograms per deciliter), alcohol consumption during pregnancy (grams/day)], father [education (≥ college graduate/< college graduate)], household [ownership of a gas stove (yes/no), annual income at time of cognitive assessment (< $40,000, $40,000–70,000, $70,000–150,000, ≥ $150,000), HOME-SF score], and neighborhood (median annual income for census tract of residence at cognitive testing). Models for third-trimester BC and PM_2.5_ exposures were also adjusted for seasonal trends (modeled as sine and cosine functions of the date of cognitive testing) (Schwartz et al. 1991).

We hypothesized that gestational age and fetal growth could be on the causal pathway between pregnancy exposures and cognitive development, so we did not adjust for these covariates in primary models, but ran sensitivity analyses adjusted for gestational age (weeks) and birth weight/gestational age *z*-score (Oken et al. 2003). We investigated the influence of individual covariates on the associations between BC exposure and verbal IQ by running a series of models adjusted for subsets of covariates.

To increase sample size (precision) and reduce bias due to missing data, we imputed missing covariates. Using a chained equation multiple imputation method (PROC MI in SAS), we generated 50 imputed data sets including all Project Viva participants with live births (*n* = 2,128) (Rubin 2004; White et al. 2011). The imputation model included all exposures, outcomes, and covariates under study, as well as additional potential predictors (White et al. 2011). In final analytic models, we combined imputed data sets using PROC MIANALYZE in SAS. Participants with missing exposure or outcome data for a given exposure–outcome analysis were excluded from that analysis.

As a sensitivity analysis, we re-ran primary models excluding participants (*n* = 21) who did not complete the full set of cognitive assessments. To assess the influence of maternal IQ and maternal and child blood lead as potential confounders, we ran primary models with and without these covariates. In addition, we assessed effect measure modification of associations with near-residence traffic density, BC, and PM_2.5_ by child sex and annual household income at assessment (< or ≥ $70,000) using interaction terms. Numbers of participants in the < 50 m category of major roadway proximity were not sufficient to assess effect modification with this exposure.

We performed analyses involving penalized splines in R version 3.0.0 (R Core Team 2013); all other analyses were completed using SAS version 9.3 (SAS Institute Inc., Cary, NC).

## Results

*Participant characteristics*. We included 1,109 children with data on at least one exposure and one outcome. Characteristics of included participants were generally similar to those of participants excluded due to missing exposure or outcome data, although excluded participants had somewhat lower birth weight (3,433 vs. 3,486 g), shorter duration of breastfeeding (4.5 vs. 6.5 months), higher rates of maternal smoking in pregnancy (16 vs. 10%), and slightly lower levels of parental education and household income (see Supplemental Material, Table S2). Distributions of covariates in the original and the imputed data sets were very similar (see Supplemental Material, Table S2). Table 1 presents distributions of participant characteristics and cognitive assessment scores. Cognitive assessment scores were generally lower among participants living closest (< 50 m) to a major roadway at birth (*n* = 34).

**Table 1:** Characteristics of study participants [*n* (%) or mean ± SD, after imputation for covariates], overall and by category of major roadway proximity at birth address.

Characteristic	Overall (*n *= 1,109)	Distance to nearest major roadway		
		≥ 200 m (*n *= 970)	50 to < 200 m (*n *= 100)	< 50 m(*n *= 34)
Cognitive assessments
Verbal IQ (KBIT-2) (*n *= 1,099)	111.8 ± 15.1	111.9 ± 15.3	112.4 ± 14.0	107.8 ± 14.1
Nonverbal IQ (KBIT-2) (*n *= 1,109)	106.3 ± 17.0	106.4 ± 16.8	108.4 ± 16.7	98.6 ± 20.5
Visual motor (WRAVMA) (*n *= 1,102)	92.0 ± 16.7	92.3 ± 16.9	90.7 ± 15.2	87.9 ± 16.9
Design memory (WRAML2) (*n *= 1,105)	8.0 ± 2.8	8.0 ± 2.8	8.1 ± 2.6	8.0 ± 2.8
Picture memory (WRAML2) (*n *= 1,105)	8.9 ± 3.0	8.9 ± 3.0	8.6 ± 2.9	8.7 ± 3.2
Child characteristics
Age at testing (years)	8.0 ± 0.8	8.0 ± 0.9	7.9 ± 0.8	7.9 ± 0.9
Sex
Female	555 (50)	477 (49)	58 (58)	17 (50)
Male	554 (50)	493 (51)	42 (42)	17 (50)
Gestational age (weeks)	39.5 ± 1.8	39.6 ± 1.8	39.5 ± 1.7	39.1 ± 2.4
Birth weight (grams)	3,486 ± 560	3,493 ± 559	3,526 ± 506	3,206 ± 679
Birth weight/gestational age *z*-score	0.19 ± 0.97	0.20 ± 0.98	0.33 ± 0.89	–0.27 ± 1.02
Duration of breastfeeding (months up to 12)	6.3 ± 4.8	6.3 ± 4.8	6.5 ± 4.7	5.5 ± 4.2
Early childhood blood lead (μg/dL)	2.3 ± 2.1	2.3 ± 2.0	2.3 ± 1.7	2.7 ± 2.6
Maternal characteristics
Age at enrollment (years)	32.1 ± 5.4	32.2 ± 5.3	31.2 ± 5.7	30.5 ± 5.7
IQ (KBIT-2 composite)	106.3 ± 15.5	106.4 ± 15.4	106.5 ± 16.6	103.7 ± 14.1
Parity
Nulliparous	528 (48)	465 (48)	42 (42)	17 (50)
1	400 (36)	341 (35)	43 (43)	15 (44)
≥ 2	181 (16)	164 (17)	15 (15)	2 (6)
Education
College degree or beyond	752 (68)	651 (67)	73 (73)	24 (71)
Less than college degree	357 (32)	319 (33)	27 (27)	10 (29)
Race/ethnicity
White	747 (67)	665 (69)	62 (62)	17 (50)
Black	181 (16)	149 (15)	23 (23)	9 (26)
Asian	59 (5)	50 (5)	5 (5)	4 (12)
Hispanic	72 (6)	62 (6)	7 (7)	2 (6)
Other	50 (5)	44 (5)	3 (3)	2 (6)
Alcohol consumption during pregnancy (g/day)	0.18 ± 0.25	0.18 ± 0.25	0.20 ± 0.29	0.15 ± 0.24
Smoking status
Smoked during pregnancy	109 (10)	94 (10)	12 (12)	3 (9)
Former smoker	214 (19)	191 (20)	16 (16)	7 (21)
Never smoker	786 (71)	685 (71)	72 (72)	24 (71)
Exposure to secondhand smoke during pregnancy
≥ 1 hr/week	189 (17)	160 (17)	22 (22)	5 (15)
< 1 hr/week	920 (83)	810 (84)	78 (78)	29 (85)
Marital/cohabitation status
Married or cohabitating	1,011 (91)	887 (91)	88 (88)	32 (94)
Not married or cohabitating	98 (9)	83 (9)	12 (12)	2 (6)
Blood lead in pregnancy (μg/dL)	1.2 ± 0.8	1.2 ± 0.8	1.3 ± 0.9	1.5 ± 1.0
Paternal characteristics
Education
College degree or beyond	699 (63)	609 (63)	63 (63)	24 (72)
Less than college degree	411 (37)	361 (37)	38 (38)	10 (29)
Household/neighborhood characteristics
Household income at mid-childhood
≤ $40,000	141 (13)	115 (12)	19 (19)	5 (16)
> $40,000 to ≤ $70,000	153 (14)	137 (14)	11 (11)	4 (13)
> $70,000 to ≤ $150,000	502 (45)	454 (47)	34 (34)	13 (37)
> $150,000	313 (28)	264 (27)	35 (35)	12 (34)
HOME-SF score	18.4 ± 2.2	18.3 ± 2.3	18.3 ± 2.1	18.8 ± 1.9
Gas stove in home at age 1 year
Yes	652 (59)	570 (59)	59 (59)	20 (60)
No	457 (41)	400 (41)	41 (41)	14 (41)
Census tract median annual household income, address at cognitive testing ($)	64,800 ± 24,733	64,743 ± 23,869	63,810 ± 28,889	73,046 ± 34,727

*Exposures*. Table 2 describes the distribution of exposures. A total of 34 participants (3%) lived within 50 m of a major roadway at birth (of these, 23 resided in urban areas, with 11 in suburban or rural areas). Spearman correlation coefficients among exposure variables are presented in the Supplemental Material, Table S3; as expected, distance to major roadway was negatively correlated with traffic density, BC, and PM_2.5_ exposures, with residences further from major roadways having lower local traffic density and lower pollutant concentrations. Third-trimester BC exposures were higher for nonwhite mothers, nulliparous mothers, and for mothers who were not married or cohabiting, had lower education, lower household income, higher exposure to secondhand smoke, or lived in households with gas stoves (see Supplemental Material, Table S4).

**Table 2:** Traffic-related pollution exposures.

Exposure	*n*	Mean ± SD or *n* (%)
Distance to nearest major roadway, birth address	1,104
< 50 m		34 (3)
50 to < 200 m		100 (9)
≥ 200 m		970 (88)
Distance to nearest major roadway, mid-childhood address	1,102
< 50 m		26 (2)
50 to < 200 m		88 (8)
≥ 200 m		988 (90)
Near-residence traffic density
Birth address (km × vehicles/day)	1,101	1,428 ± 1,850
Mid-childhood address (km × vehicles/day)	993	1,140 ± 1,620
Black carbon (BC) exposure
Third trimester (μg/m^3^)	1,095	0.69 ± 0.23
Birth–6 years (μg/m^3^)	945	0.56 ± 0.16
Year before cognitive testing (μg/m^3^)	965	0.47 ± 0.15
Fine particulate (PM_2.5_) exposure
Third trimester (μg/m^3^)	960	12.3 ± 2.6
Birth–6 years (μg/m^3^)	975	11.3 ± 1.7
Year before cognitive testing (μg/m^3^)	1,036	9.4 ± 1.9

Ninety-seven percent of participants remained in the same category of roadway proximity from enrollment through the child’s birth, whereas 91% remained from birth to 3 years, and 88% remained from birth to cognitive testing. Participants in the closest road proximity category (< 50 m) were more likely to move to a different category between birth and cognitive testing (61% in this group changed categories, vs. 56% for the 50 to < 200 m group, and 6% for the ≥ 200 m group).

**Figure 1:**
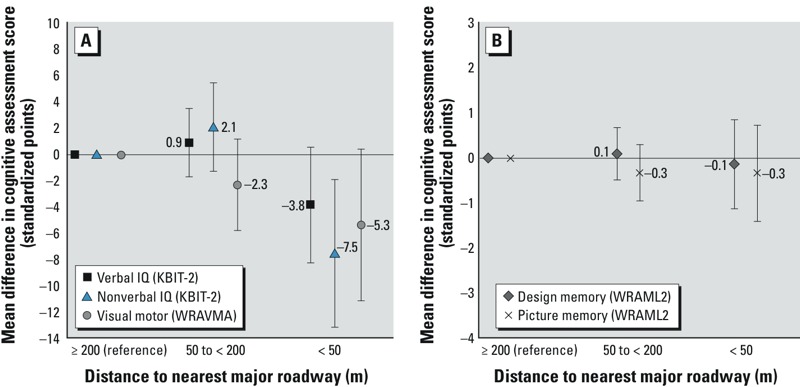
Mean differences (95% CIs) in cognitive assessment scores associated with residential proximity to major roadway at birth. (*A*) Results for standardized cognitive assessment scores scaled to mean ± SD = 100 ± 15 (KBIT-2 verbal and nonverbal IQ; WRAVMA visual motor). (*B*) Results for standardized cognitive assessment scores scaled to mean ± SD = 10 ± 3 (WRAML2 design memory and picture memory). All models were adjusted for characteristics of child (age, sex, breastfeeding duration, early childhood blood lead), mother (age, parity, race/ethnicity, education, IQ, marital/cohabitation status, and blood lead, smoking, secondhand smoke exposure, and alcohol in pregnancy), father (education), household (income, home caretaking environment, gas stove), and neighborhood (census tract median income).

*Major roadway proximity*. In fully adjusted regression models, children with birth addresses within 50 m of a major roadway had lower mid-childhood nonverbal IQ scores [–7.5 points; 95% confidence interval (CI): –13.1, –1.9], verbal IQ scores (–3.8 points; 95% CI: –8.2, 0.6), and visual motor scores (–5.3 points; 95% CI: –11.0, 0.4) than participants living ≥ 200 m from a major roadway (Figure 1A). Design memory and picture memory scores did not differ substantially by major roadway proximity (Figure 1B). Estimated associations were similar in minimally adjusted models (see Supplemental Material, Table S5). Nonverbal IQ scores were also lower among children living < 50 m versus ≥ 200 m from a major roadway at the time of cognitive testing (–5.6 points; 95% CI: –11.9, 0.8), but living 50 to < 200 m versus ≥ 200 m at cognitive testing predicted higher nonverbal IQ (3.2 points; 95% CI: –0.4, 6.8). Other cognitive scores did not appear to differ across levels of mid-childhood major roadway proximity (see Supplemental Material, Table S6).

*Near-residence traffic density*. Children with higher near-residence traffic density at birth had slightly higher nonverbal IQ, visual motor, design memory, and picture memory scores (Table 3). Traffic density at cognitive testing predicted lower verbal IQ in minimally adjusted models (–1.3 points per IQR increase; 95% CI: –2.5, –0.1) (see Supplemental Material, Table S5), but fully adjusted models suggested associations with higher verbal IQ (1.1 points per IQR increase; 95% CI: 0.0, 2.2) and nonverbal IQ (see Supplemental Material, Table S6). Traffic density at mid-childhood address did not appear associated with other cognitive outcomes.

**Table 3:** Mean differences (95% CIs) in cognitive assessment scores associated with IQR increases in prenatal near-residence traffic density, black carbon, and fine particulate matter exposure.*^a^*

Exposure	Verbal IQ (KBIT-2)^*b*^	Nonverbal IQ (KBIT-2)^*b*^	Visual motor (WRAVMA)^*b*^	Design memory (WRAML2)^*c*^	Picture memory (WRAML2)^*c*^
Ln(near-residence traffic density at birth address)	0.2 (–0.3, 0.8)	0.8 (0.1, 1.6)	0.7 (–0.1, 1.5)	0.1 (0.0, 0.2)	0.1 (0.0, 0.2)
Third-trimester BC	0.2 (–0.9, 1.3)	1.3 (–0.2, 2.7)	0.9 (–0.6, 2.4)	–0.1 (–0.3, 0.2)	–0.1 (–0.3, 0.2)
Third-trimester PM_2.5_	–0.2 (–1.4, 1.1)	–0.2 (–1.8, 1.4)	0.9 (–0.8, 2.5)	–0.1 (–0.3, 0.2)	0.1 (–0.2, 0.4)
Abbreviations: BC, black carbon; IQR, interquartile range; PM_2.5_, fine particulate matter. IQR = 1.6 ln(km × vehicles/day) for traffic density at birth, 0.32 μg/m^3^ for third-trimester BC, 3.8 μg/m^3^ for third-trimester PM_2.5_.^***a***^All models were adjusted for characteristics of child (age, sex, breastfeeding duration, early childhood blood lead), mother (age, parity, race/ethnicity, education, IQ, marital/­cohabitation status, and blood lead, smoking, secondhand smoke exposure, and alcohol in pregnancy), father (education), household (income, home caretaking environment, gas stove), and neighborhood (census tract median income). BC and PM_2.5_ models were also adjusted for seasonal trends. ^***b***^KBIT-2 and WRAVMA scores were standardized to mean ± SD = 100 ± 15. ^***c***^WRAML2 scores were standardized to mean ± SD = 10 ± 3.					

*Black carbon (BC)*. Third-trimester BC exposure predicted lower verbal IQ in minimally adjusted models (–1.9 points per IQR increase; 95% CI: –3.2, –0.7) (see Supplemental Material, Table S5). Fully adjusted results, however, showed no association between third-trimester BC exposure and verbal IQ (Table 3). Similarly, in minimally adjusted models, BC exposure in the year before cognitive testing was associated with lower verbal IQ (–2.4 points per IQR increase; 95% CI: –3.6, –1.1), as was birth–6 years BC exposure (–2.6 points per IQR increase; 95% CI: –3.9, –1.4), but after full adjustment, each predicted slightly higher verbal IQ (year before cognitive testing: 1.1 points per IQR increase; 95% CI: –0.2, 2.4; birth–6 years: 0.9; 95% CI: –0.4, 2.2) (see Supplemental Material, Tables S5 and S6). The attenuation or reversal of direction in these associations appeared to be driven by adjustment for sociodemographic variables, specifically maternal race/ethnicity and census-tract median income (see Supplemental Material, Figure S1).

Third-trimester BC and birth–6 years BC were also associated with higher nonverbal IQ in fully adjusted models (third trimester: 1.3 points per IQR increase; 95% CI: –0.2, 2.7; birth–6 years: 1.7; 95% CI: 0.1, 3.4) (Table 3; see also Supplemental Material, Table S6). Neither third trimester, birth–6 years, nor year before testing BC appeared associated with other cognitive outcomes.

*Fine particulate matter (PM_2.5_)*. In fully adjusted models, there was no evidence of adverse association between third-trimester PM_2.5_ and cognitive outcomes; an IQR increase in third-trimester PM_2.5_ predicted slightly higher nonverbal IQ (0.9 points; 95% CI: –0.8, 2.5) (Table 3). In minimally adjusted models, an IQR increase in birth–6 years or year before testing PM_2.5_ predicted lower verbal IQ (birth–6 years: –1.9 points; 95% CI: –3.0, –0.8; year before testing: –1.0 points; 95% CI: –2.2, 0.2) (see Supplemental Material, Table S5), but predicted slightly higher verbal IQ in fully adjusted models (birth–6 years: 0.7 points; 95% CI: –0.4, 1.7; year before testing: 1.1 points; 95% CI: 0.0, 2.2) (see Supplemental Material, Table S6). Birth–6 years PM_2.5_ was also associated with small increases in nonverbal IQ (1.1 points per IQR increase; 95% CI: –0.2, 2.5) and visual motor skills (1.8 points per IQR increase; 95% CI: 0.4, 3.2) in fully adjusted models (see Supplemental Material, Table S6).

*Sensitivity analyses*. Variance inflation factors were < 2 for all covariates included in primary models, suggesting that collinearity among covariates did not reduce precision of effect estimates. Visual inspection suggested that penalized splines for the continuous exposure–outcome relationships generally did not deviate substantially from linearity (data not shown). For all exposures, periods, and outcomes, results generated by models additionally adjusted for birth outcomes (considered as potential mediators), were similar to those from primary models (see Supplemental Material, Table S7). Analyses excluding participants with incomplete cognitive assessments (*n* = 21) also yielded similar results (data not shown). Results of models excluding maternal IQ and maternal and child blood lead were very similar to those of primary models (data not shown). We did not observe consistent patterns of effect measure modification by sex or household income (data not shown).

## Discussion

Among children residing primarily in urban and suburban Eastern Massachusetts, prenatal residential proximity to major roadways (< 50 m) predicted lower nonverbal intelligence, verbal intelligence, and visual motor abilities in mid-childhood. These findings are based on observations from a small number of children (*n* = 34) living < 50 m from a major roadway at birth, and should therefore be interpreted cautiously, but suggest that major roadway proximity during gestation may be associated with decrements in function across a range of cognitive domains. The 7.5-point (95% CI: –13.1, –1.9) decrement in nonverbal IQ associated with residence at birth < 50 m compared with ≥ 200 m from a major roadway is similar in scale to the decrement in Full Scale IQ associated with an increase in childhood blood lead from 2.4 to 30 μg/dL (the 5th to the 95th percentile) in an international pooled analysis (6.9 points) (Lanphear et al. 2005).

Two of the cognitive outcomes related to prenatal major roadway proximity in our study (verbal and nonverbal intelligence) were inversely associated with exposure to PAHs measured in late pregnancy among 5-year-olds in New York City and Poland (Edwards et al. 2010; Perera et al. 2009). Although we know of no previous reports of associations of early-life residential roadway proximity with cognitive outcomes in children, prenatal proximity to freeways was associated in another study with development of autism in childhood (Volk et al. 2011).

Living < 50 m from a major roadway at the time of cognitive testing predicted a smaller decrease in nonverbal IQ than roadway proximity at birth, and did not appear associated with other cognitive outcomes. These findings suggest that major roadway proximity during gestation and early life might have a greater influence on cognitive development than major roadway proximity later in childhood, but associations at both time periods were very imprecise due to small numbers of participants in the closest proximity group.

The associations we observed between major roadway proximity and child cognition may be attributable to tailpipe emissions, but may also involve other roadway-related exposures such as road dust or noise, or associated neighborhood characteristics such as walkability and access to green space. Findings may also reflect random error or bias, particularly given the small numbers of observations in the closest proximity group, and require replication in other populations. Air pollution exposure could impair neurodevelopment through several pathways, including endocrine disruption, epigenetic changes, or systemic inflammatory responses leading to oxidative stress (Calderón-Garcidueñas et al. 2011; Edwards et al. 2010; Perera et al. 2006; Volk et al. 2011). There is also evidence that chronic exposure to noise may be associated with decreased cognitive function in children (Stansfeld et al. 2005).

Although we observed evidence of associations of prenatal, birth–6 years, and proximal exposure to BC with verbal IQ in minimally adjusted models, associations were attenuated or reversed following adjustment for covariates (in particular, maternal race and census tract median income), suggesting that spatial relationships in this study population between traffic-related pollution exposures and sociodemographic factors strongly related to cognitive outcomes may limit our ability to determine whether there are independent effects of these pollution measures on these outcomes; prenatal and childhood exposure to BC also tended to predict somewhat higher nonverbal IQ in fully adjusted models. The correlation observed between pollution estimates and sociodemographic variables is likely attributable to complex geographical covariation, partly arising from clustering of sociodemographic characteristics and pollution in communities. We have not been able to adequately define this clustering; doing so would likely require new statistical methodology and additional variables and observations. We are therefore unable to completely disentangle the influences of pollutants and socioeconomic conditions on cognition. Conversely, however, we did not observe evidence of substantial confounding in the models of prenatal major roadway proximity by any of the measured covariates, suggesting that residual confounding by sociodemographic factors is unlikely to explain the observed major roadway proximity–cognition associations.

Our results are not consistent with a prior report from another Boston cohort of lower verbal IQ, nonverbal IQ, and visual memory scores among children with higher childhood exposure to BC (Suglia et al. 2008). Exposure levels in our study were similar to those in the other cohort, but the inconsistency in findings may stem from differences in population demographics between the studied cohorts, or differences in analytical approaches.

Limitations in the spatial resolution of one important input to the PM_2.5_ model—the satellite aerosol optical depth data—may have constrained our ability to detect associations between PM_2.5_ exposure and cognition; advances in remote sensing technology should increase precision of estimates in the future. It is also possible that exposure to particular components of PM_2.5_ might have an influence on cognition that we were unable to measure when estimating associations with PM_2.5_ total mass.

Our observation of associations of lower cognitive scores with major roadway proximity but not with traffic density may stem from imprecision in our estimates of traffic density or uncaptured variability in the traffic pollution sources represented. In addition, in contrast to our roadway proximity exposure, which included the category < 50 m, our traffic density estimates included all traffic within 100 m of a residence, and important constituents of traffic-related pollution, such as ultrafine particles, may be concentrated nearer to roadways than 100 m (Karner et al. 2010; Zhu et al. 2002).

Because numerous studies have demonstrated modest decrements in birth weight and gestation length with greater air pollution exposure (Stieb et al. 2012) and lower fetal growth and shorter gestation length are inversely associated with cognitive development (Shenkin et al. 2004; Yang et al. 2010), we hypothesized that preterm birth or intrauterine growth restriction might lie on the causal pathway between prenatal traffic exposure and cognition. However, the associations we observed between major roadway proximity at birth and cognitive outcomes in primary models did not change appreciably in sensitivity analyses adjusted for gestational age and birth weight for gestational age *z*-score, suggesting that relationships between major roadway proximity and cognitive development were not mediated by these factors.

Although we examined multiple exposures and outcomes, these measures were correlated, and we drew conclusions from patterns across results rather than individual significant associations, so the issue of multiple comparisons is not of serious concern. Additional limitations in our study should be noted, however. To capture the potential effects of exposure to air pollutants that exist in high concentrations only very near to major roadways, we delineated our closest category of major roadway proximity at < 50 m. Because a relatively small number of participants resided in this category, our effect estimates for this group had limited precision. Although we worked to minimize error in geocoding, exposure misclassification due to positional error and time–activity patterns of participants is still possible (Lane et al. 2013). Because most participants remained in the same category of major roadway proximity throughout the study period, it is also possible that prenatal distance to roadway may represent cumulative exposure before and after pregnancy.

Because of early loss to follow-up in Project Viva, which is typical of longitudinal birth cohort studies, our study included only 52% of the original cohort. Although it is reassuring that early-life exposures and other characteristics of included participants were generally similar to those of the excluded participants, there were small differences in some factors correlated with both exposures and outcomes, suggesting that selection bias is possible. Finally, although our study population was reasonably diverse in terms of race/ethnicity and income, study participants all had health insurance coverage and access to early prenatal care, and were on average relatively well off and highly educated, so generalizability of results to less advantaged populations may be limited.

This is one of the first studies to assess relationships between neurodevelopment and exposure to traffic-related pollution across periods in gestation and childhood in an effort to identify particularly vulnerable developmental windows. We included multiple cognitive assessments representing several domains of brain function. Our analyses were strengthened by the capacity to examine the influence of potential confounders including maternal IQ, maternal and child blood lead, smoking and secondhand smoke exposure, and multiple measures related to household and area-level socioeconomic status.

## Conclusion

Our findings suggest that prenatal proximity to major roadways may negatively influence performance across a range of cognitive domains in mid-childhood. Proximity of children’s homes to major roadways at the time of cognitive testing appeared less strongly predictive of cognitive performance, suggesting that gestation (or early life) could be a more sensitive period than mid-childhood to the effects of major roadway proximity on cognitive development. Because only a small number of study participants lived very close to major roadways, however, estimated associations with roadway proximity were imprecise; our findings should therefore be considered preliminary and require replication in other populations. Prenatal and childhood near-residence traffic density, BC, and PM_2.5_ did not display consistent patterns of association with child cognition.

## Supplemental Material

(508 KB) PDF
