# Supplementary material for: Prenatal and Childhood Traffic-Related Pollution Exposure and Childhood Cognition in the Project Viva Cohort (Massachusetts, USA)
Source: Environ Health Perspect. 2015 Apr 3;123(10):1072–8. doi: 10.1289/ehp.1408803 (PMC4590752; doi:10.1289/ehp.1408803)
Supplement: (508 KB) PDF [file ehp.1408803.s001.acco.pdf]

Environ Health Perspect
doi: 10.1289/ehp.1408803

Note to Readers: *EHP* strives to ensure that all journal content is accessible to all readers. However, some figures and Supplemental Material published in *EHP* articles may not conform to 508 standards due to the complexity of the information being presented. If you need assistance accessing journal content, please contact ehp508@niehs.nih.gov. Our staff will work with you to assess and meet your accessibility needs within 3 working days.

Supplemental Material

Prenatal and Childhood Traffic-Related Pollution Exposure and Childhood Cognition in the Project Viva Cohort (Massachusetts, USA)

Maria H. Harris, Diane R. Gold, Sheryl L. Rifas-Shiman, Steven J. Melly, Antonella Zanobetti, Brent A. Coull, Joel D. Schwartz, Alexandros Gryparis, Itai Kloog, Petros Koutrakis, David C. Bellinger, Roberta F. White, Sharon K. Sagiv, and Emily Oken

Table of Contents

Table S1. Neurobehavioral functional domains measured by cognitive assessments.

Table S2. Characteristics of study subjects with and without imputation of covariates, and enrolled subjects excluded from the present study due to missing exposure or outcome data.

Table S3. Spearman correlation coefficients among continuous exposure variables.

Table S4. Third trimester black carbon exposure across levels of covariates.

Table S5. Minimally adjusted model results for mean differences in cognitive assessment scores associated with major roadway proximity and interquartile range increases in near-residence traffic density, black carbon (BC) and fine particulate matter ($PM_{2.5}$) exposure (95% confidence intervals).

Table S6. Mean differences in cognitive assessment scores associated with childhood major roadway proximity, and interquartile range increases in near-residence traffic density, black carbon (BC) and fine particulate matter ($PM_{2.5}$) exposure (95% confidence intervals).

Table S7. Mean differences in cognitive assessment scores associated with major roadway proximity at birth and interquartile range increases in near-residence traffic density at birth, third trimester BC and third trimester fine $PM_{2.5}$ (95% confidence intervals), adjusted for full set of covariates and potential mediators.

Figure S1. Associations (+ 95% confidence intervals) between verbal IQ score[a] and black carbon exposure with staged adjustment for influential covariates[b]. IQR, interquartile range. IQR: third trimester=0.32 $\mu g/m^3$, birth–age 6=0.22 $\mu g/m^3$, year before cognitive testing= 0.20 $\mu g/m^3$. [a]KBIT-2 score standardized to mean=100, standard deviation=15. [b]Model 0 adjusted for child sex and age. Model 1 adjusted for characteristics of child (age, sex, breastfeeding duration, early childhood blood lead), mother (age, parity, race/ethnicity, education, IQ, marital/cohabitation status, and blood lead, smoking, secondhand smoke exposure, and alcohol in pregnancy), father (education), household (income, home caretaking environment, gas stove) and neighborhood (census tract median income). All third trimester models also adjusted for seasonal trends.

References

Table S1. Neurobehavioral functional domains measured by cognitive assessments.

Domain	Verbal IQ (KBIT-2)	Non-Verbal IQ (KBIT-2)	Visual Motor (WRAVMA)	Design Memory (WRAML2)	Picture Memory (WRAML2)
Crystallized intelligence[a]	●	●			
Word knowledge	●				
Executive function	●	●		●	●
Fine motor skills			●		
Visuospatial perception		●	●	●	
Visual memory				●	●

[a]"[A]ccessible stores of knowledge, and the ability to acquire further knowledge via familiar learning strategies" (Wasserman 2005).

Table S2. Characteristics of study subjects with and without imputation of covariates, and enrolled subjects excluded from the present study due to missing exposure or outcome data.

Characteristic	N, observations for which missing data was imputed	Mean ± SD or %, study participants, with imputed covariates (n=1,109)	Mean ± SD or %, study participants, without imputation (n=1,109)	Mean ± SD or %, excluded Project Viva participants (n=1,019)
**Exposures**				
Proximity to major roadway at birth address	-			
<50 m	-	3	3	4
50–<200 m	-	9	9	9
≥200 m	-	88	88	88
Near-residence traffic density				
Birth address (km*vehicles/day)	-	1,428 ± 1,850	1,428 ± 1,850	1,563 ± 2,330
Black carbon (BC) exposure				
Third trimester (μg/m^3)	-	0.69 ± 0.23	0.69 ± 0.23	0.70 ± 0.25
Fine particulate ($PM_{2.5}$) exposure				
Third trimester (μg/m^3)	-	12.3 ± 2.6	12.3 ± 2.6	12.2 ± 2.4
**Cognitive Assessments**				
KBIT-2 – Verbal IQ	-	111.8 ± 15.1	111.8 ± 15.1	-
KBIT-2 – Non-Verbal IQ	-	106.3 ± 17.0	106.3 ± 17.0	-
WRAVMA – Visual Motor	-	92.0 ± 16.7	92.0 ± 16.7	-
WRAML2 – Design Memory	-	8.0 ± 2.8	8.0 ± 2.8	-
WRAML2 – Picture Memory	-	8.9 ± 3.0	8.9 ± 3.0	-
**Child characteristics**				
Age at testing (years)	0	8.0 ± 0.9	8.0 ± 0.8	-
Sex	0			
Female (%)	-	50	50	53
Male (%)	-	50	50	47
Gestational age (weeks)	0	39.5 ± 1.8	39.5 ± 1.8	39.3 ± 2.2
Birth weight (grams)	1	3,486 ± 559	3,486 ± 560	3,433± 625
Birth weight/gestational age z-score	1	0.19 ± 0.97	0.19 ± 0.97	0.15 ± 0.97
Duration of breastfeeding (months up to 12)	81	6.3 ± 4.8	6.5 ± 4.6	4.5 ± 4.3
Early childhood blood lead (μg/dL)	690	2.3 ± 2.1	2.2 ± 1.4	2.5 ±1.8
**Maternal characteristics**				
Age at enrollment (years)	0	32.1 ± 5.3	32.1 ± 5.4	31.5 ± 5.1

Characteristic	N, observations for which missing data was imputed	Mean ± SD or %, study participants, with imputed covariates (n=1,109)	Mean ± SD or %, study participants, without imputation (n=1,109)	Mean ± SD or %, excluded Project Viva participants (n=1,019)
IQ (KBIT-2 composite)	21	106.3 ± 15.5	106.3 **±** 15.4	-
Parity	0			
Nulliparous (%)	-	48	48	48
1 (%)	-	36	36	35
≥2 (%)	-	16	16	17
Education	11			
College degree or beyond (%)	-	68	68	61
Less than college degree (%)	-	32	32	39
Race/ethnicity	6			
White (%)	-	67	68	65
Black (%)	-	16	16	17
Asian (%)	-	5	5	6
Hispanic (%)	-	6	6	8
Other (%)	-	5	5	3
Alcohol consumption during pregnancy (g/day)	124	0.18 ± 0.25	0.18 **±** 0.24	0.19 **±** 0.26
Smoking status	4			
Smoked during pregnancy (%)	-	10	10	16
Former smoker (%)	-	19	19	18
Never smoker (%)	-	71	71	66
Exposure to secondhand smoke during pregnancy	117			
≥1 hour per week (%)	-	17	16	16
<1 hour per week (%)	-	83	84	84
Marital/cohabitation status	7			
Married or cohabitating (%)	-	91	91	92
Not married or cohabitating (%)	-	9	9	8
Blood lead in pregnancy (µg/dL)	538	1.2 ± 0.8	1.2 ± 0.6	1.3 ± 0.8
**Paternal characteristics**				
Education	112			
College degree or beyond (%)	-	63	67	62
Less than college degree (%)	-	37	33	38
**Household/neighborhood characteristics**				
Household income at mid-childhood	70			

Characteristic	N, observations for which missing data was imputed	Mean ± SD or %, study participants, with imputed covariates (n=1,109)	Mean ± SD or %, study participants, without imputation (n=1,109)	Mean ± SD or %, excluded Project Viva participants (n=1,019)
≤$40 K (%)	-	13	12	-
$≥40–<70 K (%)	-	14	14	-
$>70–≤150 K (%)	-	45	46	-
>$150 K (%)	-	28	29	-
Household income at birth	-			
≤$40 K (%)	-	-	15	17
$>40–≤70 K (%)	-	-	21	26
>$70 K (%)	-	-	64	58
HOME-SF score	34	18.4 ± 2.2	18.4 **±** 2.2	-
Gas stove in home at age 1	230			
Yes (%)	-	59	58	54
No (%)	-	41	42	46
Census tract median annual household income, address at cognitive testing ($)	6	64,800 ± 24,678	64,902 ± 24,759	-
Census tract median annual household income, address at birth ($)	-	-	56,905 ± 21,607	57,124 ± 21,113

Table S3. Spearman correlation coefficients among continuous exposure variables.[a]

Exposure	Distance to major roadway (birth)	Distance to major roadway (mid-childhood)	Near-residence traffic density (birth)	Near-residence traffic density (mid-childhood)	BC (3rd trimester)	BC (birth–age 6)	BC (year before testing)	$PM_{2.5}$ (3rd trimester)	$PM_{2.5}$ (birth–age 6)	$PM_{2.5}$ (year before testing)
**Near-residence traffic density**										
Birth address (km*vehicles/day)	-0.35	-0.22	-	0.54	0.49	0.49	0.39	0.31	0.35	0.17
Mid-childhood address (km*vehicles/day)	-0.23	-0.40	0.54	-	0.39	0.60	0.65	0.28	0.45	0.37
**Black carbon (BC) exposure**										
Third trimester ($\mu g/m^3$)	-0.31	-0.25	0.49	0.39	-	0.71	0.58	0.52	0.44	0.23
Birth–age 6 ($\mu g/m^3$)	-0.29	-0.38	0.49	0.60	0.71	-	0.88	0.38	0.61	0.39
Year before cognitive testing ($\mu g/m^3$)	-0.25	-0.40	0.39	0.65	0.58	0.88	-	0.33	0.54	0.45
**Fine particulate ($PM_{2.5}$) exposure**										
Third trimester ($\mu g/m^3$)	-0.29	-0.18	0.31	0.28	0.52	0.38	0.33	-	0.59	0.27
Birth–age 6 ($\mu g/m^3$)	-0.29	-0.34	0.35	0.45	0.44	0.61	0.54	0.59	-	0.64
Year before cognitive testing ($\mu g/m^3$)	-0.19	-0.27	0.17	0.37	0.23	0.39	0.45	0.27	0.64	-

[a]All correlation coefficient p-values <.0001.

Table S4. Third trimester black carbon exposure across levels of covariates.

Characteristic	Mean ± SD third trimester black carbon exposure (μg/m^3)
**Maternal characteristics**	
Parity	
Nulliparous (%)	0.71 ± 0.23
1 (%)	0.68 ± 0.23
≥2 (%)	0.68 ± 0.24
Education	
College degree or beyond (%)	0.67 ± 0.23
Less than college degree (%)	0.75 ± 0.22
Race/ethnicity	
White (%)	0.65 ± 0.23
Black (%)	0.79 ± 0.18
Asian (%)	0.73 ± 0.22
Hispanic (%)	0.78 ± 0.21
Other (%)	0.81 ± 0.21
Smoking status	
Smoked during pregnancy (%)	0.71 ± 0.23
Former smoker (%)	0.66 ± 0.25
Never smoker (%)	0.70 ± 0.23
Exposure to secondhand smoke during pregnancy	
≥1 hour per week (%)	0.74 ± 0.23
<1 hour per week (%)	0.68 ± 0.24
Marital/cohabitation status	
Married or cohabitating (%)	0.68 ± 0.23
Not married or cohabitating (%)	0.81 ± 0.19
**Paternal characteristics**	
Education	
College degree or beyond (%)	0.66 ± 0.24
Less than college degree (%)	0.74 ± 0.23
**Household/neighborhood characteristics**	
Household income at mid-childhood	
≤$40K (%)	0.77 ± 0.21
$>40–≤70K (%)	0.76 ± 0.22
$>70–≤150K (%)	0.68 ± 0.24
>$150 K (%)	0.65 ± 0.23
Gas stove in home at age 1	
Yes (%)	0.73 ± 0.19
No (%)	0.65 ± 0.31

Table S5. Minimally adjusted model results for mean differences in cognitive assessment scores associated with major roadway proximity and interquartile range increases in near-residence traffic density, black carbon (BC) and fine particulate matter ($PM_{2.5}$) exposure (95% confidence intervals).[a]

Exposure	Verbal IQ (KBIT-2)[b]	Non-Verbal IQ (KBIT-2)[b]	Visual Motor (WRAVMA)[b]	Design Memory (WRAML2)[c]	Picture Memory (WRAML2)[c]
**Proximity to major roadway at birth address**					
<50 m	-4.3 (-9.5, 0.9)	-7.8 (-13.6, -2.0)	-4.6 (-10.3, 1.1)	0.0 (-0.9, 1.0)	-0.2 (-1.3, 0.8)
50–<200 m	0.0 (-3.0, 3.1)	1.8 (-1.6, 5.3)	-2.1 (-5.5, 1.3)	0.1 (-0.5, 0.7)	-0.4 (-1.0, 0.3)
≥200 m	0.0 (Reference)	0.0 (Reference)	0.0 (Reference)	0.0 (Reference)	0.0 (Reference)
**Proximity to major roadway at mid-childhood address**					
<50 m	-2.8 (-8.7, 3.1)	-7.7 (-14.2, -1.1)	-0.5 (-7.0, 5.9)	0.1 (-1.0, 1.2)	0.2 (-1.0, 1.4)
50–<200 m	0.0 (-3.3, 3.3)	2.9 (-0.7, 6.6)	-1.7 (-5.4, 1.9)	-0.2 (-0.8, 0.4)	0.3 (-0.4, 0.9)
≥200 m	0.0 (Reference)	0.0 (Reference)	0.0 (Reference)	0.0 (Reference)	0.0 (Reference)
**Ln(near-residence traffic density)**					
Birth address	-0.3 (-0.9, 0.4)	0.6 (-0.2, 1.3)	0.5 (-0.3, 1.2)	0.1 (-0.1, 0.2)	0.1 (0.0, 0.2)
Mid-childhood address	-1.3 (-2.5, -0.1)	-0.1 (-1.5, 1.2)	-0.5 (-1.9, 0.8)	0.0 (-0.3, 0.2)	-0.1 (-0.3, 0.2)
**BC exposure**					
Third trimester	-1.9 (-3.2, -0.7)	0.2 (-1.2, 1.6)	0.1 (-1.3, 1.5)	-0.2 (-0.4, 0.1)	-0.1 (-0.4, 0.1)
Birth–age 6	-2.6 (-3.9, -1.4)	-0.3 (-1.8, 1.1)	-0.4 (-1.9, 1.0)	-0.2 (-0.4, 0.0)	-0.2 (-0.4, 0.1)
Year before behavioral assessment	-2.4 (-3.6, -1.1)	-1.0 (-2.4, 0.5)	-1.0 (-2.4, 0.4)	-0.2 (-0.4, 0.0)	-0.1 (-0.3, 0.2)
**$PM_{2.5}$ exposure**					
Third trimester	-0.8 (-2.3, 0.6)	-0.1 (-1.7, 1.5)	0.6 (-1.0, 2.1)	-0.1 (-0.4, 0.1)	0.1 (-0.2, 0.4)
Birth–age 6	-1.9 (-3.0, -0.8)	-0.3 (-1.6, 0.9)	0.7 (-0.6, 1.9)	-0.3 (-0.5, -0.1)	0.0 (-0.2, 0.3)
Year before behavioral assessment	-1.0 (-2.2, 0.2)	-0.4 (-1.8, 1.0)	0.3 (-1.0, 1.7)	-0.2 (-0.4, 0.0)	-0.1 (-0.3, 0.2)

BC, black carbon; $PM_{2.5}$, fine particulate matter. Interquartile range=1.6 ln(km*vehicles/day) for traffic density at birth, 1.8 ln(km*vehicles/day) for traffic density at mid-childhood, 0.32 $\mu g/m^3$ for third trimester BC, 0.22 $\mu g/m^3$ for birth–age 6 BC, 0.20 $\mu g/m^3$ for BC in year before cognitive testing, 3.8 $\mu g/m^3$ for third trimester $PM_{2.5}$, 2.1 $\mu g/m^3$ for birth–age 6 $PM_{2.5}$, 2.5 $\mu g/m^3$ for $PM_{2.5}$ in year before cognitive testing.

[a]Adjusted for child age at cognitive testing and sex. Third trimester BC and $PM_{2.5}$ models also adjusted for seasonal trends. [b]KBIT-2 and WRAVMA scores standardized to mean=100, standard deviation=15. [c]WRAML2 scores standardized to mean=10, standard deviation=3.

Table S6. Mean differences in cognitive assessment scores associated with childhood major roadway proximity, and interquartile range increases in near-residence traffic density, black carbon (BC) and fine particulate matter ($PM_{2.5}$) exposure (95% confidence intervals).[a]

Exposure	Verbal IQ (KBIT-2)[b]	Non-Verbal IQ (KBIT-2)[b]	Visual Motor (WRAVMA)[b]	Design Memory (WRAML2)[c]	Picture Memory (WRAML2)[c]
**Proximity to major roadway at mid-childhood address**					
<50 m	1.0 (-4.0, 6.0)	-5.6 (-11.9, 0.8)	0.4 (-6.1, 6.9)	0.2 (-0.9, 1.3)	0.2 (-1.0, 1.4)
50-<200 m	1.0 (-1.8, 3.7)	3.2 (-0.4, 6.8)	-1.4 (-5.0, 2.3)	-0.2 (-0.8, 0.4)	-0.3 (-0.4, 0.9)
≥200 m	0.0 (Reference)	0.0 (Reference)	0.0 (Reference)	0.0 (Reference)	0.0 (Reference)
Ln(near-residence traffic density)	1.1 (0.0, 2.2)	1.1 (-0.4, 2.5)	0.1 (-1.4, 1.6)	0.1 (-0.1, 0.4)	0.0 (-0.3, 0.3)
BC exposure, birth–age 6	0.9 (-0.4, 2.2)	1.7 (0.1, 3.4)	0.7 (-1.0, 2.4)	-0.1 (-0.4, 0.2)	-1.0 (-0.4, 0.2)
BC exposure, year before cognitive testing	1.1 (-0.2, 2.4)	0.7 (-0.9, 2.4)	-0.2 (-1.8, 1.5)	-0.1 (-0.4, 0.2)	0.0 (-0.3, 0.3)
$PM_{2.5}$ exposure, birth–age 6	0.7 (-0.4, 1.7)	1.1 (-0.2, 2.5)	1.8 (0.4, 3.2)	-0.2 (-0.4, 0.1)	0.1 (-0.1, 0.4)
$PM_{2.5}$ exposure, year before cognitive testing	1.1 (0.0, 2.2)	0.7 (-0.8, 2.1)	1.0 (-0.5, 2.5)	-0.1 (-0.4, 0.1)	0.0 (-0.2, 0.3)

BC, black carbon; $PM_{2.5}$, fine particulate matter. Interquartile range=1.8 ln(km*vehicles/day) for traffic density at mid-childhood, 0.22 μg/m^3 for birth–age 6 BC, 0.20 μg/m^3 for BC in year before cognitive testing, 2.1 μg/m^3 for birth–age 6 $PM_{2.5}$, 2.5 μg/m^3 for $PM_{2.5}$ in year before cognitive testing.

[a]All models adjusted for characteristics of child (age, sex, breastfeeding duration, early childhood blood lead), mother (age, parity, race/ethnicity, education, IQ, marital/cohabitation status, and blood lead, smoking, secondhand smoke exposure, and alcohol in pregnancy), father (education), household (income, home caretaking environment, gas stove) and neighborhood (census tract median income). [b]KBIT-2 and WRAVMA scores standardized to mean=100, standard deviation=15. [c]WRAML2 scores standardized to mean=10, standard deviation=3.

Table S7. Mean differences in cognitive assessment scores associated with major roadway proximity at birth and interquartile range increases in near-residence traffic density at birth, third trimester BC and third trimester fine $PM_{2.5}$ (95% confidence intervals), adjusted for full set of covariates and potential mediators.[a]

Exposure	Verbal IQ (KBIT-2)[c]	Non-Verbal IQ (KBIT-2)[c]	Visual Motor (WRAVMA)[c]	Design Memory (WRAML2)[d]	Picture Memory (WRAML2)[d]
Proximity to major roadway at birth address					
<50 m	-3.6 (-8.0, 0.8)	-7.3 (-12.9, -1.7)	-5.4 (-11.2, 0.4)	-0.1 (-1.1, 0.9)	-0.4 (-1.5, 0.6)
50–<200 m	0.8 (-1.8, 3.4)	2.1 (-1.3, 5.4)	-2.2 (-5.7, 1.3)	0.1 (-0.5, 0.7)	-0.3 (-0.9, 0.3)
≥200 m	0.0 (Reference)	0.0 (Reference)	0.0 (Reference)	0.0 (Reference)	0.0 (Reference)
Ln(Near-residence traffic density at birth address)	0.3 (-0.3, 0.8)	0.8 (0.1, 1.6)	0.7 (-0.1, 1.5)	0.1 (0.0, 0.2)	0.1 (-0.1, 0.2)
Third trimester BC	0.2 (-0.9, 1.4)	1.2 (-0.2, 2.7)	0.9 (-0.6, 2.4)	-0.1 (-0.3, 0.2)	-0.1 (-0.3, 0.2)
Third trimester $PM_{2.5}$	-0.1 (-1.3, 1.2)	-0.2 (-1.8, 1.5)	0.8 (-0.8, 2.5)	-0.1 (-0.3, 0.2)	0.1 (-0.2, 0.4)

BC, black carbon; $PM_{2.5}$, fine particulate matter. Interquartile range =1.6 ln(km*vehicles/day) for traffic density at birth, 0.32 $\mu g/m^3$ for third trimester BC, 3.8 $\mu g/m^3$ for third trimester $PM_{2.5}$.

[a]Models adjusted for characteristics of child (age, sex, breastfeeding duration, early childhood blood lead), mother (age, parity, race/ethnicity, education, IQ, marital/cohabitation status, and blood lead, smoking, secondhand smoke exposure, and alcohol in pregnancy), father (education), household (income, home caretaking environment, gas stove) and neighborhood (census tract median income) + gestational age (in weeks) and birth weight/gestational age z-score. BC and $PM_{2.5}$ models also adjusted for seasonal trends. [b]KBIT-2 and WRAVMA scores standardized to mean=100, standard deviation=15. [c]WRAML2 scores standardized to mean=10, standard deviation=3.

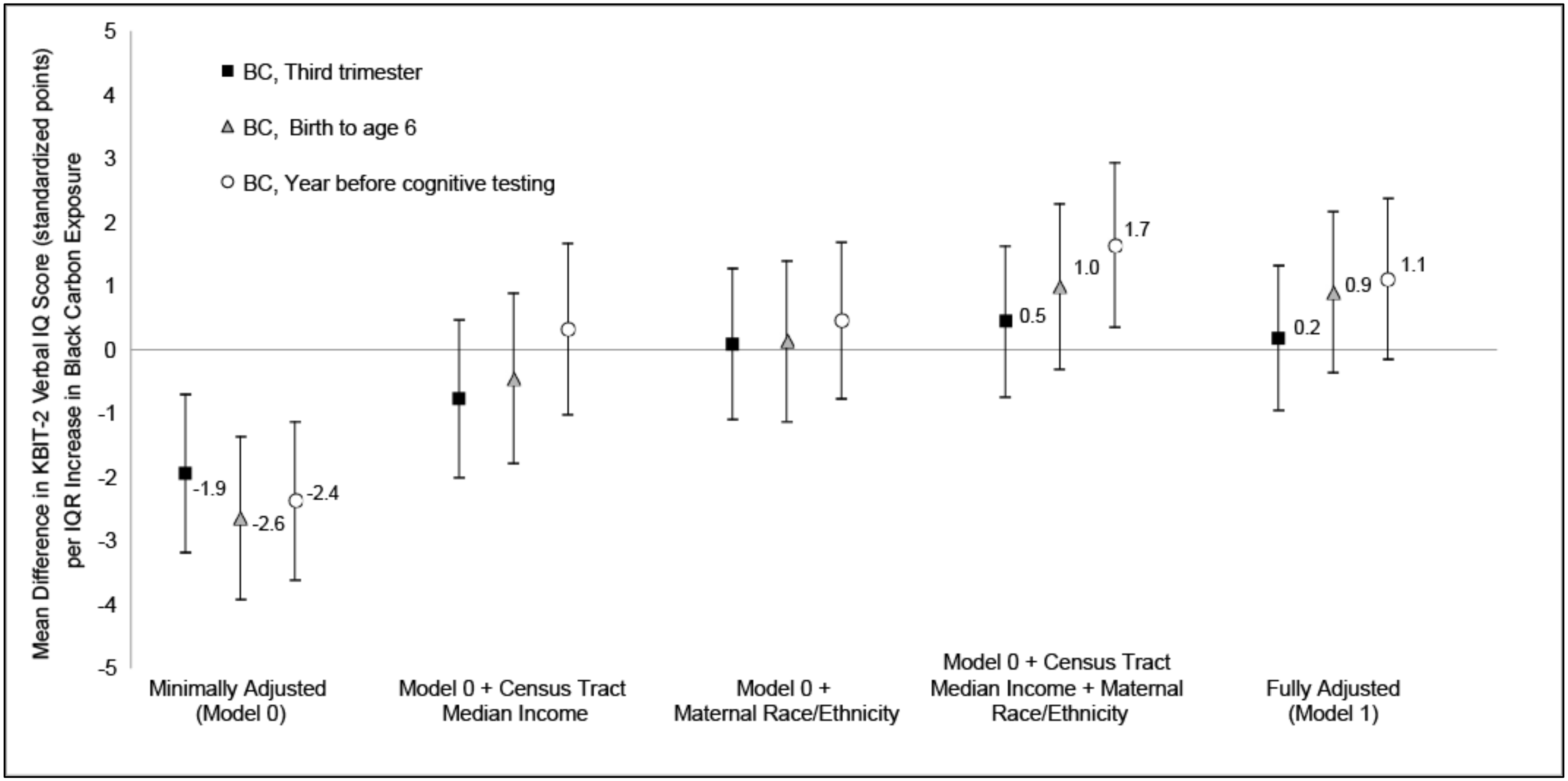

Figure S1. Associations (+ 95% confidence intervals) between verbal IQ score[a] and black carbon exposure with staged adjustment for influential covariates[b]. IQR, interquartile range. IQR: third trimester=0.32 μg/m^3, birth−age 6=0.22 μg/m^3, year before cognitive testing= 0.20 μg/m^3. [a]KBIT-2 score standardized to mean=100, standard deviation=15. [b]Model 0 adjusted for child sex and age. Model 1 adjusted for characteristics of child (age, sex, breastfeeding duration, early childhood blood lead), mother (age, parity, race/ethnicity, education, IQ, marital/cohabitation status, and blood lead, smoking, secondhand smoke exposure, and alcohol in pregnancy), father (education), household (income, home caretaking environment, gas stove) and neighborhood (census tract median income). All third trimester models also adjusted for seasonal trends.

References

Wasserman JD. 2005. A History of Intelligence Assessment: The Unfinished Tapestry. In: Contemporary Intellectual Assessment: Theories, Tests, and Issues. (Flanagan DP and Harrison PL, eds.). New York: Guilford Press, 3-70.
